# Transposon mutagenesis and genome sequencing identify two novel, tandem genes involved in the colony spreading of *Flavobacterium collinsii*, isolated from an ayu fish, *Plecoglossus altivelis*

**DOI:** 10.3389/fcimb.2023.1095919

**Published:** 2023-02-10

**Authors:** Yoshio Kondo, Kenichi Ohara, Ryoji Fujii, Yudai Nakai, Chikara Sato, Mariko Naito, Takayuki Tsukuba, Tomoko Kadowaki, Keiko Sato

**Affiliations:** ^1^ Department of Pediatric Dentistry, Nagasaki University Graduate School of Biomedical Sciences, Nagasaki, Japan; ^2^ Gifu Prefectural Research Institute for Fisheries and Aquatic Environments, Gifu, Japan; ^3^ Department of Frontier Oral Science, Nagasaki University Graduate School of Biomedical Sciences, Nagasaki, Japan; ^4^ School of Integrative and Global Majors (SIGMA), University of Tsukuba, Ibaraki, Japan; ^5^ Biological Science Course, Graduate School of Science and Engineering, Aoyama Gakuin University, Kanagawa, Japan; ^6^ Division of Immune Homeostasis, Department of Pathology and Microbiology, Nihon University School of Medicine, Tokyo, Japan; ^7^ Division of Microbiology, Department of Pathology and Microbiology, Nihon University School of Medicine, Tokyo, Japan; ^8^ Department of Microbiology and Oral Infection, Nagasaki University Graduate School of Biomedical Sciences, Nagasaki, Japan; ^9^ Department of Dental Pharmacology, Nagasaki University Graduate School of Biomedical Sciences, Nagasaki, Japan

**Keywords:** *Flavobacterium collinsii*, *Flavobacterium johnsoniae*, colony spreading, gliding motility, type IX secretion system, biofilm formation, glycosylation

## Abstract

Bacteria of the family *Flavobacteriaceae* (flavobacteria) primarily comprise nonpathogenic bacteria that inhabit soil and water (both marine and freshwater). However, some bacterial species in the family, including *Flavobacterium psychrophilum* and *Flavobacterium columnare*, are known to be pathogenic to fish. Flavobacteria, including the abovementioned pathogenic bacteria, belong to the phylum *Bacteroidota* and possess two phylum-specific features, gliding motility and a protein secretion system, which are energized by a common motor complex. Herein, we focused on *Flavobacterium collinsii* (GiFuPREF103) isolated from a diseased fish (*Plecoglossus altivelis*). Genomic analysis of *F. collinsii* GiFuPREF103 revealed the presence of a type IX secretion system and additional genes associated with gliding motility and spreading. Using transposon mutagenesis, we isolated two mutants with altered colony morphology and colony spreading ability; these mutants had transposon insertions in *pep25* and *lbp26*. The glycosylation material profiles revealed that these mutants lacked the high-molecular-weight glycosylated materials present in the wild-type strain. In addition, the wild-type strains exhibited fast cell population movement at the edge of the spreading colony, whereas reduced cell population behavior was observed in the *pep25-* and *lbp26*-mutant strains. In the aqueous environment, the surface layers of these mutant strains were more hydrophobic, and they formed biofilms with enhanced microcolony growth compared to those with the wild-type. In *Flavobacterium johnsoniae*, the *Fjoh_0352* and *Fjoh_0353* mutant strains were generated, which were based on the ortholog genes of *pep25* and *lbp26*. In these *F. johnsoniae* mutants, as in *F. collinsii* GiFuPREF103, colonies with diminished spreading capacity were formed. Furthermore, cell population migration was observed at the edge of the colony in wild-type *F. johnsoniae*, whereas individual cells, and not cell populations, migrated in these mutant strains. The findings of the present study indicate that *pep25* and *lbp26* contribute to the colony spreading of *F. collinsii.*

## 1 Introduction

Two important bacterial species belonging to genus *Flavobacterium* are responsible for the most common infectious diseases in freshwater-reared fish: *F. psychrophilum*, the etiological agent of bacterial cold-water disease (BCWD), also known as rainbow trout fry syndrome, and *F. columnare*, responsible for columnaris disease. In Japan, in 1987 an outbreak of BCWD occurred at an aquaculture farm in Tokushima Prefecture (Wakabayashi, 1994), and its occurrence in rivers was first confirmed in 1993 (Iida and Mizokami, 1996). Since then, BCWD spread across Japan, causing severe damage to the inland fishery industry (Inoue, 2000). The dynamics of BCWD in rivers has been investigated in several studies, previously (Fujiwara-Nagata et al., 2013; Fujiwara-Nagata et al., 2019). Reportedly, BCWD outbreaks are strongly influenced by water temperature, where low water temperatures are considered optimal for this pathogen (Uddin and Wakabayashi, 1997; Tenma et al., 2021). Temperature-induced stress immunity responses in *Plecoglossus altivelis* (Del Valle and Taniguchi, 1995), higher population densities (Iguchi et al., 2002; Iguchi et al., 2003), and higher concentrations of suspended solids in river water (Awata et al., 2011) also influenced the occurrence and spread of BCWD. Pathogenic traits of BCWD include necrotic lesions, partial skin darkening, exophthalmia, anemia, ascites, and vertebral deformities in fish (Bernardet, 1998; Kondo et al., 2002). These symptoms have been associated with phenotypic features of *F. psychrophilum*, such as extracellular proteases (Duchaud et al., 2007), adhesion or biofilm formation (Sundell et al., 2019), and hemolysis (Castillo et al., 2015).

*F. johnsoniae*, which belongs to *Bacteroidota*, has long been studied to understand the mechanism underlying the bacteria’s motility. These motility mechanisms differ from the motility mechanisms in cyanobacteria, *Myxococcus xanthus* (Nan et al., 2014), and *Mycoplasma mobile* (Jarrell and McBride, 2008). In *F. johnsoniae*, the cell surface adhesin SprB is rapidly propelled along a closed helical loop by the gliding motility machinery driven by a rotary motor. This results in the gliding movement of the cell (Nakane et al., 2013; Shrivastava and Berg, 2020). The gliding motility of *F. johnsoniae* is usually observed on agar medium as irregular, thin, flattened spreading colonies with feather-like edges; this phenomenon is termed as colony spreading (Stanier, 1947). The internal structure of *F. johnsoniae* colony spreading consists of cells embedded in a self-produced extracellular polymeric matrix that contains a filamentous network and vesicles, indicating biofilm formation (Sato et al., 2021a).

Genetic studies focusing on mechanisms underlying gliding motility and colony spreading ability found that in *F. johnsoniae* the *gld* and *spr* genes are involved in gliding motility and colony spreading, respectively, while the *rem* gene is redundant (Agarwal et al., 1997; Braun et al., 2005; Rhodes et al., 2011b; Shrivastava et al., 2013). Studies on *F. johnsoniae* and the non-motile periodontal bacterium *Porphyromonas gingivalis* revealed that Gld and Spr homologous proteins were components of a protein secretion system (McBride and Zhu, 2013). This system has been termed as type IX secretion system (T9SS) and is specific to the phylum *Bacteroidota* (Sato et al., 2010; McBride and Zhu, 2013). T9SS has been previously reported to be associated with virulence and gliding motility. Recently, it has been reported that deletion of *gldN*, a T9SS component, in *F. columnare* or *F. psychrophilum* inhibits protein secretion, compromises the gliding motility, and abolishes the pathogenicity in zebrafish and rainbow trout (Li et al., 2017; Barbier et al., 2020). Studies on human oral pathogens belonging to *Bacteroidota* have revealed that a T9SS also contributes to the secretion of virulence factors (Sato et al., 2013; Narita et al., 2014; Kondo et al., 2018; Naito et al., 2022).

The *F. johnsoniae* T9SS secretes cell surface adhesins and other proteins, such as SprB, required for gliding motility (Sato et al., 2010; Shrivastava et al., 2013; Kharade and McBride, 2014). Proteins secreted by the T9SS have a conserved C-terminal domain called the CTD signal that allows protein to cross the outer membrane (Seers et al., 2006; Chen et al., 2011; Veith et al., 2013). The two types of CTD signals are type A and type B; they differ in some of the T9SS components, required for their export (Kulkarni et al., 2017). Many of the secreted proteins of *P. gingivalis* have several copies of type A CTD proteins. Sortase PorU removes the cell surface type A CTD signals and replaces it with anionic lipopolysaccharide (A-LPS), which anchors the CTD protein to the cell surface *via* covalent linkage to the A-LPS (Glew et al., 2012; Gorasia et al., 2015; Glew et al., 2017). In contrast, SprB, the dominant motility adhesin in *F. johnsoniae*, was reported to have a T9SS secreted type B CTD (Kulkarni et al., 2019). Recently, it was suggested that type B CTD proteins bind to PorP-like proteins for anchoring to the cell surface (Gorasia et al., 2022).

In some members of the phylum *Bacteroidota*, protein glycosylation is involved in anchoring T9SS cargo proteins to the bacterial cell surface and causing their virulence (Veith et al., 2020; Gorasia et al., 2020). The lack of *fpgA* gene predicted to encode a type-2 glycosyltransferase in *F. psychrophilum* was associated to pleiotropic changes, including the loss of colony spreading (Pérez-Pascual et al., 2015). However, not much is known about the contribution of glycosylation modifications to gliding motility. In this study, we isolated *Flavobacterium* sp. from an ayu *Plecoglossus altivelis* with BCWD in an aquaculture farm of Gifu Prefecture. *F. collinsii* GiFuPREF103 is more closely related to the *F. johnsoniae* than to the BCWD pathogen, *F. psychrophilum*. In this study, using the transposon mutagenesis, we isolated *Flavobacterium* sp. GiFuPREF103 mutants that formed less-spreading colonies. Two of these mutants, designated as FTN25 and FTN26, had an insertion in *pep25* and *lpb26* that encoded a putative lipopolysaccharide biosynthesis protein and polysaccharide export protein, respectively. The phenotypic analysis of two mutants was performed to study the gliding motility, colony spreading, and biofilm formation of this strain.

## 2 Materials and methods

### 2.1 Bacterial strain and colony cultivation

The details of the bacterial strains used herein are shown in **Table 1** (Simon and Puhler, 1983; Rhodes et al., 2011a). *Flavobacterium collinsii* GiFuPREF103 was grown in modified Cytophaga (MCP) medium (Wakabayashi and Egusa, 1974) at 20°C. To culture erythromycin-resistant *F. collinsii* GiFuPREF103 strains, erythromycin (100 μg/mL) was added to the medium. *Flavobacterium johnsoniae* strains were grown in casitone yeast extract medium at 25°C. For the selection and maintenance of antibiotic-resistant *F. johnsoniae* strains, antibiotics were added to the medium at the following concentrations: streptomycin, 100 μg/mL and erythromycin, 100 μg/mL. To observe colony spreading, *F. collinsii* GiFuPREF103 or *F. johnsoniae* cells were grown in MCP medium at 20°C or 25°C with shaking (175 rpm) overnight, respectively. The cells were centrifuged at 800 × *g* for 10 min at 20°C. The pellet was resuspended in the washing buffer (10 mM Tris–HCl pH 7.4) by vortexing, and the suspension was centrifuged at 800 × *g* for 10 min at 20°C. The process was repeated twice. The cells were spotted onto MCP agar medium (agar; BD BACTO Agar, Becton, Dickinson and Co., Franklin Lakes, NJ, USA) in a 3 or 9 cm dish.

**Table 1:** Bacterial strains and plasmids used in this study.

Strain	Description	Ref. or source
*E. coli* strain		
XL1-Blue	Strain used for general cloning	Stratagene (La Jolla, CA, USA)
S17-1λpir	RP4-2-Tc::Mu *aph*::Tn7 *recA*, Sm^r^	(Simon and Puhler, 1983)
*F. collinsii* GiFuPREF103		this study
FTN25	*F. collinsii* GiFuPREF103 *Flavo103_17390*::*ermF*/ITR, Em^r^	this study
FTN26	*F. collinsii* GiFuPREF103 *Flavo103_17400*::*ermF*/ITR, Em^r^	this study
*F. johnsoniae* strain		
CJ1827	*rpsL2*, Sm^r^ “wild-type” *F.johnsoniae* strain used in construction of deletion mutants	(Rhodes et al., 2011a)
CJ1922	rpsL2 Δ*sprB*, Sm^r^	(Rhodes et al., 2011a)
FLG001	*rpsL2* Δ*Fjoh_0352*, Sm^r^	this study
FLG002	*rpsL2* Δ*Fjoh_0353*, Sm^r^	this study
*Flavobacterium* spp. Plasmid		
pMI07	Delivery vector for *ermF/*ITR transposon in Bacteroides species.	(Ichimura et al., 2014)
pRR51	suicide vector	(Rhodes et al., 2011a)
pFG001	Em^r^, pRR51 containing *ermF*/ITR of pMI07	this study
pNS1	Ap^r^ Em^r^, *E. coli-F. johnsoniae* shuttle plasmid	(Imamura et al., 2018)
pFJ29	Ap^r^ Em^r^, Expression vector carrying with *ompA* promoter and *gfp*	(Chen et al., 2007)
pFG002	Ap^r^ Em^r^, *pNS1-Fjoh_0352-gfp*	this study
pFG003	Ap^r^ Em^r^, *pNS1-Fjoh_0353-gfp*	this study

### 2.2 16S rRNA sequence analysis and identification of bacterial strain

Bacterial strains that formed yellow colonies on MCP agar medium and had the ability to disperse were isolated from *Plecoglossus altivelis* with BCWD. Each strain cell was used as a template and PCR was performed using primer pair 27F & 1500R to amplify the 16S rRNA region. The PCR products were sequenced using primers 27F, 1500R, 800F, and 800R.

### 2.3 Genome sequencing of *F. collinsii* GiFuPREF103

MCP broth was inoculated with *F. collinsii* GiFuPREF103 and incubated under aerobic conditions. Genomic DNA was extracted using a Wizard Genomic DNA Purification Kit (Promega, Madison, WI, USA) according to the manufacturer’s instructions. The library was prepared using MGIEasy FS DNA Library Preparation Set, MGIEasy Circularization Kit, and DNBSEQ-G400 High-throughput Sequencing Set (MGI Tech Co., Shenzhen, China), according to the manufacturer’s instructions. The sample was sequenced using the DNBSEQ-G400 system (MGI Tech Co.) with 2 × 150-bp reads. Subsequent *de novo* assembly using the Spades (ver. 3.13.2) protocol yielded 189 contigs. Each contig sequence was annotated using DFAST and deposited as *F. collinsii* GiFuPREF103 in the DDBJ/EMBL/GenBank database under the accession number BOVI01000001–BOVI01000189 (**Table S1**).

### 2.4 Phylogenetic tree

The 16S rRNA phylogenetic tree was constructed using the neighbor-joining method with the MEGA tool (ver. 11). DNA sequences of 16S rRNA of *Flavobacterium* spp. were obtained from the National Center for Biotechnology Information database (**Table S2**).

### 2.5 Transposon mutagenesis and identification of disrupted genes

For the construction of the *ermF*/ITR delivery vector in *Flavobacterium* spp., the pMI07 plasmid was digested using SalI and SphI and inserted into the SalI-SphI region of pRR51 to yield pFG001. The plasmid pFG001 was introduced into *F. collinsii* GiFuPREF103 through conjugation with *Escherichia coli* S17-1λpir, as previously described (Rhodes et al., 2011a). An erythromycin-resistant transconjugant was obtained by plating cells on MCP agar containing erythromycin. Transformants were screened for non-spreading colony formation. The insertion sites of *ermF*/ITR in non-spreading colony formation mutants were identified through nested arbitrarily primed polymerase chain reaction (AP-PCR) as previously described (Ichimura et al., 2014). The genomes of mutants were used as templates for AP-PCR. First round of PCR was performed using a random primer (AR8) and mariner-A. Second round PCR was performed using the primer AR2 and mariner-B. The product was subsequently purified and sequenced using the sequence primer mariner-S.

### 2.6 *F. johnsoniae* strains, plasmids, and mutant construction

The details of plasmids used herein are shown in **Table 1** (Chen et al., 2007; Rhodes et al., 2011a; Ichimura et al., 2014; Imamura et al., 2018).

*F. johnsoniae* gene deletion mutants were constructed as follows: DNA regions upstream and downstream of a gene were PCR-amplified from the chromosomal DNA of *F. johnsoniae.* The primers used herein were gene-UF-BamHI plus gene-UR-SalI and gene-DF-SalI plus gene-DR-SphI, respectively, where ‘U’ indicates upstream, ‘F’ indicates forward, ‘D’ indicates downstream, and ‘R’ indicates reverse. The primers used herein are listed in **Table S3**. The amplified DNA was cloned into the pGEM-T Easy vector (Promega). The upstream region was digested using BamHI and SalI. The downstream DNA was digested with SalI and SphI. Both the digested products were ligated using pRR51 that was digested with BamHI and SphI. The plasmid was introduced into *F. johnsoniae* CJ1827 *via* triparental conjugation and deletion mutants were isolated as previously described (Rhodes et al., 2011a).

For construction of an *F. johnsoniae* strain expressing Fjoh_0352-green fluorescent protein (GFP) and Fjoh_0353-GFP, the *F. johnsoniae* chromosomal genes encoding *Fjoh_0352* and *Fjoh_0353* were PCR-amplified using the primer pair Fjoh0352-coF-BamHI & Fjoh0352-GR-NotI and Fjoh0353-coF-BamHI & Fjoh0353-GR-NotI, respectively. The amplified DNA was digested with BamHI and NotI and inserted into the corresponding region of pNS1, resulting in the plasmid pNS1 containing *Fjoh_0352-gfp* (pFG002) and *Fjoh_0353-gfp* (pFG003). Plasmids pFJ29, pFG002, and pFG003 were introduced into *F. johnsoniae* CJ1827, FLG001, and FLG002, respectively, through triparental conjugation, and GFP-expressing cells were isolated from each strain, as previously reported (Rhodes et al., 2011a).

### 2.7 Localization of Pep25 and Lbp26

Cells were examined using microscopy to identify Pep25 and Lbp26 on the cell membrane. *F. johnsoniae* expressing Δ*fjoh_0352*/fjoh_0352-GFP or Δ*fjoh_0353*/fjoh_0353-GFP (200 μL) were fixed using 1% formaldehyde for 3 min at 25°C on a glass slide. After washing twice with phosphate-buffered saline (PBS), DNA and cell membranes were detected by incubating with 1/500 dilution of 4′,6-diamidino-2-phenylindole (DAPI) (Invitrogen, Waltham, MA, USA) and FM4-64 (Invitrogen) 30 min. After incubation, *F. johnsoniae* cells were subsequently washed twice with PBS and examined under an Olympus IX81 microscope (Olympus, Tokyo, Japan). Images were visualized with a phase contrast objective LUCPlanFLN 100× (Olympus) and captured with a monochrome CoolSNAPHQ digital camera (Photometrics, Tucson, AZ, USA) using MetaMorph software version 6.1 (Molecular Devices, San Jose, CA, USA).

### 2.8 Subcellular fractionation

Bacterial cells from a 200-ml culture were collected, suspended in 10 mM Tris-HCl pH 7.5 and vortexed. After centrifugation at 20,000 x g for 10 min at 4°C, the supernatant, containing cell surface materials fraction, was collected. The pellet of cells was resuspended in 20 ml of PBS containing 0.1 mM *Na*-*p*-tosyl-L-lysine chloromethyl ketone (TLCK), 0.1 mM leupeptin, 25 µg/ml DNase I and 25 µg/ml RNase A disrupted using a French pressure cell with two passes at 100 MPa. The remaining intact bacterial cells were removed by centrifugation at 2,400 x *g* for 10 min, and the supernatant was subjected to ultracentrifugation at 100,000 x *g* for 60 min. The supernatant, containing the cytoplasm and periplasm fraction, was retained. The pellets, containing the membrane fraction, was retained. These sample were subjected to sodium dodecyl-sulfate polyacrylamide gel electrophoresis (SDS-PAGE).

### 2.9 Preparation of antisera

The polyclonal antiserum against SprB of *F. johnsoniae* was prepared by immunizing rabbits (Eve Bioscience, Wakayama, Japan) with a peptide derived from the amino acid sequence C^3627^NGGSNGTIKVTLGAGNTD^3645^. In the peptide, a cysteine residue was synthesized at the N-terminus of the peptide and conjugated to keyhole limpet hemocyanin (Sigma Genosys, The Woodlands, TX, USA).

### 2.10 Immunoblot analysis

Proteins separated on SDS-PAGE (3%–10%, gradient gel [ATTO]) gels were electroblotted onto a PVDF membrane (Sequi-Blot PVDF Membrane, Bio-Rad). The blots were incubated in blocking buffer containing TBS, 0.5% Tween-20, and 5% skim milk (Becton, Dickinson and Company). Subsequently, the blot was incubated with anti-SprB (diluted in blocking buffer) at 4°C overnight. Proteins were probed with the HRP-conjugated anti-rabbit immunoglobulins (Dako) (diluted in blocking buffer), visualized using the Luminata Forte Western HRP substrate (Merck Millipore), and finally detected using enhanced chemiluminescence (GE Healthcare).

### 2.11 Detection of glycoproteins on SDS-PAGE gel using Pro-Q Emerald 300

Samples were subjected to SDS-PAGE and stained with Coomassie Brilliant Blue. Glycoprotein bands were visualized using Pro-Q Emerald 300 Glycoprotein Gel and Blot Stain Kit per manufacturer’s instruction (Invitrogen, Thermo Fisher Scientific).

### 2.12 Detection of surface localized SprB on *F. johnsoniae* cells using immunofluorescence microscopy

To identify cell-surface localized SprB, *F. johnsoniae* wild-type and mutant cells were examined using immunofluorescence microscopy. Cells were incubated overnight in MCP medium at 25°C. Ten microliters of cells were diluted in 140 μL of MCP and fixed with 1% formaldehyde for 15 min. Cells were washed three times with PBS (200 μL) and blocked with 0.1% BSA in PBS for 30 min. After incubation, cells were re-incubated with of purified anti-SprB (200 μL, 1:200 dilution of 0.1% BSA in PBS) for 90 min. Cells were washed five times with PBS (200 μL) and incubated with secondary antibody conjugated to Alexa 488 (Invitrogen) and DAPI (200 μL, 1:5,000 dilution of 0.1% BSA in PBS). Cells were incubated for 3 h in the dark. After incubation, cells were washed five times with PBS and observed using a BZ-X800 microscope (KEYENCE).

### 2.13 Microscopic observations of gliding motility on a glass surface

Wild-type and mutant cells were examined for movement using phase contrast microscopy. Cells were cultured overnight in MCP medium at 20°C on a shaker, as described previously. Tunnel slides were prepared using double sided tape, glass microscope slides, and glass coverslips. Cells in growth medium were introduced into the tunnel slide, incubated for 3 min, and motility was observed with a phase contrast microscope. Images were recorded using a CoolSNAPHQ camera (Photometrics) and analyzed using MetaMorph software version 6.1 (Molecular Devices) and ImageJ version 1.53k. Rainbow traces of cell movements were made using ImageJ and the Color FootPrint macro. The average velocity for each strain was calculated from any 100 individuals. Data were analyzed for statistical significance using Student’s unpaired t-test and *p*-value of <0.05 was used as threshold for significance.

### 2.14 Time-lapse videos

Time-lapse videos were used to observe the colony margins, as previously reported (Sato et al., 2021b). The plate was inverted on the sample stage and observed from underneath using an All-in-One Fluorescence Microscope BZ-X800 (KEYENCE). Images were visualized with a phase contrast objective LUCPlanFLN 20× (Olympus). The phase contrast microscope images were taken every 30 s. The images were analyzed, adjusted, and cropped using a BZ-X800 analyzer software (KEYENCE).

Time-lapse videos were made around the colony spreading of GFP-expressing *F. johnsoniae* strains using a BX50F microscope (Olympus). The culture plate was placed on a sample stage, and a cover glass was carefully placed over the colony. After the top surface of the dendrites formed was imaged using phase contrast microscopy, fluorescence signals in the same area were observed to track the cells using confocal laser scanning fluorescent microscopy (CLSM) with an objective lens of UPlanFl 100× (Olympus) and ANDOR iXon EMCCD camera (Oxford Instruments, Abingdon, UK) in combination with Andor iQ3 software (Oxford Instruments). Exposure time of each image was typically 100 ms (excitation light: 490–510 nm, emission light: 520–550 nm). Fluorescence images were taken every 3 s for 10 min, and movies were produced.

### 2.15 Measurement of auto-aggregation

Bacterial cells were grown overnight in MCP broth at 20°C and then adjusted to an OD_595_ of 0.6. The medium was allowed to stand for 10 min to allow the bacteria to settle. The following formula was used to quantify the auto-aggregating property: 100 – (mean OD_595_ of supernatant/0.6) × 100% (Sorroche et al., 2012). Data were analyzed for statistical significance using Student’s unpaired t-test and *p*-value of <0.05 was used as threshold for significance. Significant difference tests were performed using a student-t test based on the results of three independent experiments per sample.

### 2.16 Hydrophobicity assays

Hydrophobicity was assessed as previously described (Nakao et al., 2012). Bacterial cells were grown overnight in MCP broth and standardized at OD_595_ = 1 in PBS. They were then placed into a polyethylene tube, hexadecane was added, and the tube was vigorously vortexed and subsequently incubated for 10 min at RT to allow for phase separation before the OD_595_ of the lower aqueous phase was measured. The percent hydrophobicity was calculated using the following formula: % hydrophobicity = [1 − (OD_595_ after vortexing/OD_595_ before vortexing)] ×100. Significant difference tests were performed using a student-t test based on the results of four independent experiments per sample.

### 2.17 Hemagglutination

Overnight cultures of *F. collinsii* GiFuPREF103 strains in MCP broth were centrifuged, washed with PBS, and suspended in PBS at an OD _540_ = 0.5. The bacterial suspensions were then diluted in a 2-fold series with PBS. A 100-µl aliquot of each suspension was mixed with an equal volume of defibrinated lacked chicken erythrocyte suspension (1% in PBS) and incubated in a round-bottom microtiter plate at room temperature for 3 h.

### 2.18 Biofilm observation

Three hundred microliters of 100-fold diluted overnight culture was added to a 24-well assay plate and incubated at 20°C for 24 h. Prior to fluorescence microscopy, the supernatant of the biofilm containing suspended cells was removed and 2 mL of fresh medium was added. DAPI was used to visualize cells in the biofilm. For this purpose, cells were stained with DAPI solution in 2 mL of fresh medium, incubated at room temperature for at least 60 min, and then washed twice with 2 mL of fresh medium. Images were acquired at an excitation wavelength of 360 nm and emission wavelength of 460 nm. Fluorescently labeled lectins were used to visualize extracellular polymeric matrix (EPM) in the biofilm. Prior to addition to the biofilm, fluorescein-bound concanavalin A (Sigma-Aldrich), which binds to α-mannopyranosyl and α-glucopyranosyl residues, was added for a final concentration of 50 µg/mL; the excitation and emission wavelengths of fluorescein-conjugated ConA were 494 and 518 nm, respectively. After incubation, the biofilm was washed with fresh medium to remove excess label, and images were taken at an excitation wavelength of 470 nm and emission wavelength of 525 nm. Images were recorded on an All-in-One Fluorescence Microscope BZ-X800 (KEYENCE) and the image data were processed using a BZ-X800 Analyzer software (KEYENCE).

### 2.19 MS analysis and database search for protein identification

A gel plug containing proteins was subjected to the following procedures: washing with 50% (vol/vol) acetonitrile, washing with 100% acetonitrile, reduction with 10 mM DTT, alkylation with 55 mM iodoacetamide, washing/dehydration with 50% (vol/vol) acetonitrile, and digestion for 10 h with 10 μg/mL trypsin. The resulting peptides were extracted from the gel plug with 0.1% (vol/vol) trifluoroacetic acid/50% (vol/vol) acetonitrile and concentrated using C-18 ZipTips (Merck Millipore). Digests were spotted on a MALDI target using α–cyano-4-hydroxycinnamic acid as a matrix. Spectra were acquired on an autoflex max TOF/TOF system (Bruker). MS/MS spectra were acquired automatically. Proteins were identified using the Mascot search engine (Matrix Science, London, UK).

## 3 Results

### 3.1 *F. collinsii* GiFuPREF103 and mutagenesis through transposon insertion

**Figure 1:**
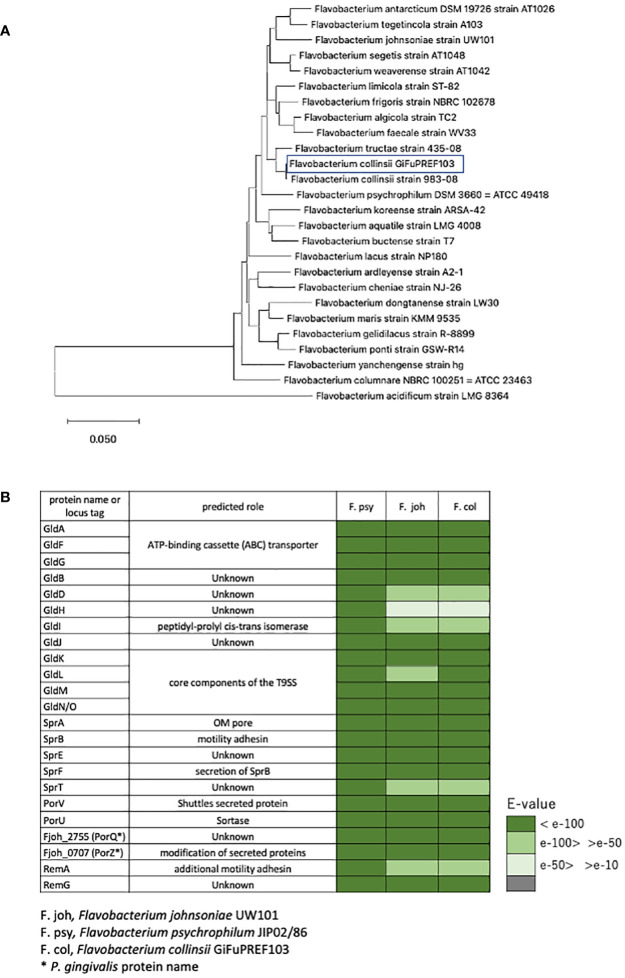
Identification of bacterial strain and the components of T9SS. **(A)** Phylogenetic tree based on 16S rRNA gene sequence of *F. collinsii* GiFuPREF103 and some strains of the genus *Flavobacterium*. The phylogenetic tree was constructed using the neighbor-joining method. Bars represent changes of 0.05 per base position. **(B)** The gliding motility protein and type IX secretion system protein were examined for their existence in the annotated genomes of *F. collinsii* GiFuPREF103.

Outbreaks of BCWD in ayu *Plecoglossus altivelis* have occurred frequently in rivers or aquaculture farm of Gifu prefecture Japan, since the first detection of *F. psychrophilum* in 2002. *Plecoglossus altivelis* with BCWD displayed ulcers on the lower jaw, ulcerative lesions on the body surface, and anemia. Bacterial strains that form yellow colonies on MCP agar medium and have colony-spreading ability were isolated from *Plecoglossus altivelis* with BCWD. The 16S rRNA sequence analysis revealed that these strains were either *Flavobacterium* sp. including *F. psychrophilum*, or *Chryseobacterium*. We focused on GiFuPREF103, a strain isolated from the gills of *Plecoglossus altivelis*, that allowed transposon insertion mutants to be created. A genomic homology search using DFAST (https://dfast.ddbj.nig.ac.jp/) revealed that GiFuPREF103 was 98.00% homologous to *Flavobacterium collinsii* CECT7796. Therefore, strain GiFUPREF103 was designated as *F. collinsii* GiFuPREF103. *F. collinsii* GiFuPREF103 was a strain phylogenetically closer to the soil bacterium *F. johnsoniae* than to *F. psychrophilum*, known fish disease bacteria (**Figure 1A**). The genome information revealed that not only the genes encoding the T9SS components (*porK*, *porL*, *porM*, porN/O, *sprA*, *sprE*, and s*prT*), which has been studied in *F. johnsoniae* and *P. gingivalis*, but also the genes related to gliding motility (*gldA*, *gldB*, *gldD*, *gldF*, *gldG*, *gldH*, *gldI*, and *gldJ*) and motility adhesin (*sprB*) were conserved in *F. collinsii* GiFuPREF103 (**Figure 1B** and **Table S4**). The genes related to protein secretion in *P. gingivalis* (*porU*, *porV*, *porQ*, and *porZ*) were also identified in *F. collinsii* GiFuPREF103. We also identified 38 carboxy-terminal domain (CTD) proteins, which are predicted secreted proteins of T9SS, and 15 *porP-like* genes including *sprF* in *F. collinsii* GiFuPREF103 (**Table S5**) (McBride, 2019).

### 3.2 Pep25 and Lbp26 are involved in colony spreading and glycosylation of materials

**Figure 2:**
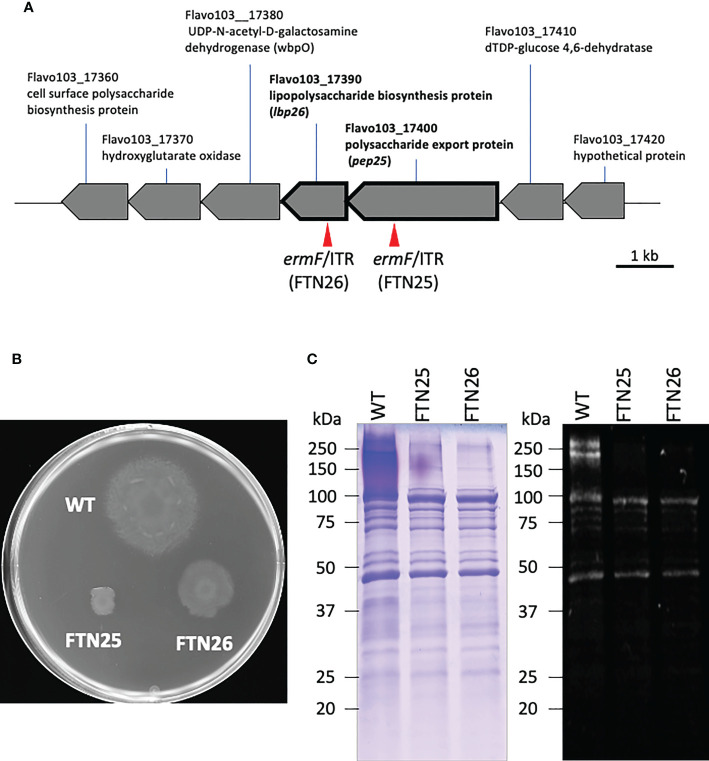
Map of the transposon insertion site and properties of FTN25 and FTN26 mutants. **(A)** Physical maps around the *pep25* and *lbp26* gene regions. Red arrow heads indicate the transposon insertion sites in FTN25 and FTN26 strains. **(B)** *F collinsii* GiFuPREF103: wild-type, FTN25 and FTN26 colonies grown on MCP agar medium at 20°C for 2 d. **(C)** SDS-PAGE and staining of surface proteins extracted from *F*. *collinsii* GiFuPREF103 wild-type, FTN25, and FTN26 strains. Extracted surface proteins were separated on 3%–10% gradient and stained with Coomassie blue (left panel) and Pro-Q Emerald (right panel) specific for protein glycosylation. The fractional marker location is indicated on the left side of each image. SDS-PAGE of the bacterial surface proteins revealed broad bands with molecular weights ranging from 270 kDa to >770 kDa in wild-type cells, whereas these were absent in FTN25 and FTN26. The Pro-Q Emerald glycan staining method revealed that the broad bands seen in wild-type cells were glycosylated materials.

Using transposon mutagenesis in *F. collinsii* GiFuPREF103, we isolated two independent mutants with altered colony morphology on MCP agar medium. An AP-PCR was performed to analyze the insertion site of *ermF*/ITR in each mutant (Ichimura et al., 2014). Sequencing of the PCR products revealed that the insertion sites were located 286 bp and 1812 bp downstream of the first nucleotide residues of the initiation codons of Flavo103_17390 and Flavo103_17400 in strains FTN26 and FTN25, respectively (**Figure 2A**). The gene cluster containing *Flavo103_17390* and *Flavo103_17400* was also conserved in *F. johnsoniae* (**Figure S1**). Flavo103_17400 showed 85% amino acid sequence similarity with Fjoh_0353 (polysaccharide export protein) of *F. johnsoniae* UW101. Thus, we named *Flavo103_17400* as *pep25*. Flavo103_17390 showed 70% amino acid sequence similarity with Fjoh_0352 (lipopolysaccharide biosynthesis protein) of *F. johnsoniae* UW101 and has a Wzz motif (PF02706.18) in the N-terminal region. Thus, we named *Flavo103_17390* as *lbp26*. FTN25 and FTN26 mutants showed less colony spreading ability than wild-type (**Figure 2B**).

To elucidate the roles of Pep25 and Lbp26 proteins, we analyzed the profiles of glycosylated material of wild-type, FTN25, and FTN26 strains. SDS-PAGE profiles of the bacterial surface proteins revealed broad bands with molecular weights ranging from 270 kDa to >770 kDa in wild-type cells, whereas these were absent in the FTN25 and FTN26 strains (**Figure 2C** left). We further examined if the materials observed in wild-type cells were glycosylated. The results of Pro-Q Emerald glycan staining suggested that two high-molecular-weight molecules of approximately 250 kDa were glycosylated (**Figure 2C** right). Flavo103_21710, type A CTD protein, and Flavo103_03160 were identified by mass spectrometry from cell surface proteins of FTN25 and FTN26, However, bands of >250 kDa macromolecules could not be identified by mass spectrometry (**Figure S2**).

### 3.3 *Fjoh_0352* and *fjoh_0353* mutants formed less-spreading colonies

As a substitute for *F. collinsii* GiFuPREF103, we used *F. johnsoniae*, a closely related species capable of genetic recombination, because genetic recombination methods other than transposon-introduced mutagenesis have not been established for *F. collinsii* GiFuPREF103. To understand the role of Pep25 and Lbp26, *F. johnsoniae* Δ*fjoh_0352* and Δ*fjoh_0353* deletion mutants were constructed. *fjoh_0352* and Δ*fjoh_0353* are orthologs of *pep25* and *lbp26*, respectively. The colony spreading ability of *F. johnsoniae* Δ*Fjoh_0352* and Δ*Fjoh_0353* was reduced compared to that of wild-type strain same as FTN25 and FTN26, respectively (**Figures 2B**, **S3**). To observe the localization, Fjoh_0352-GFP and Fjoh_0353-GFP fusion proteins were expressed in *F. johnsoniae* cells. Fluorescence microscopy observations indicated the presence of green fluorescence at the periphery of cells, suggesting the presence of Fjoh_0352 and Fjoh_0353 on the cell membrane (**Figure S4**). FM4-64 (red) and DAPI (blue) were used to detect cell membranes and DNA, respectively.

SprB is the first protein identified to be involved in gliding motility of the *F. johnsoniae* family and is one of the cell-surface adhesin proteins that allow the bacterial cells to adhere and glide on solid surfaces. SprB-deficient strains formed non-spreading colonies on agar. SprB of *F. johnsoniae* is a large protein with a repeat motif and molecular weight of 669 kDa (6497 amino acid residues). Although the number of motif repeats varies, other gliding *Bacteroidetes* bacteria also harbor the SprB protein. To investigate the effect of Fjoh_0352 and Fjoh_0353 on SprB expression, bacterial surface proteins of *F. johnsoniae* were extracted and examined through SDS-PAGE (**Figure S5A**). Immunoblot analysis revealed bands in the lanes of *F. johnsoniae* wild-type, Δ*fjoh_0352*, and Δ*fjoh_0353*, which were not observed in Δ*sprB* (**Figure S5B**). Anti-SprB immuno-staining and fluorescence microscopy revealed that SprB was located at the surface of *F. johnsoniae* wild-type, Δ*fjoh_0352*, and Δ*fjoh_0353* cells (**Figure S5C**). These results suggest that Pep25 and Lbp26 do not affect SprB expression.

### 3.4 Pep25 and Lbp26 are involved in gliding motility

**Figure 3:**
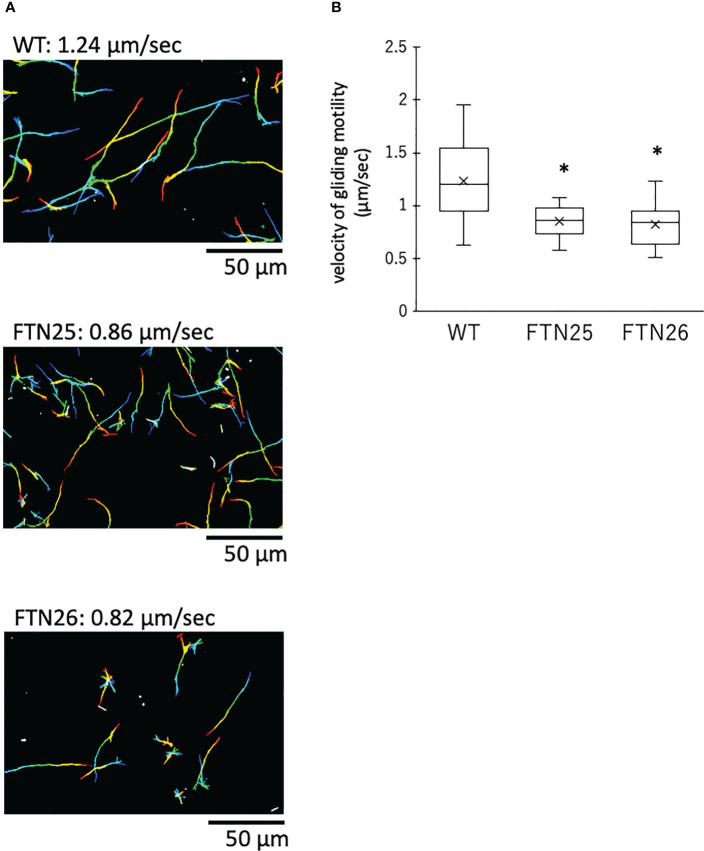
Gliding motility of *F*. *collinsii* GiFuPREF103. Bacterial cells were incubated in MCP broth overnight at 20°C with shaking, introduced into tunneled glass slides, and motility at 20°C was observed with a phase contrast microscope. **(A)** Images show the gliding motility trajectory of each bacterial cell. Cell location of each frame was colored from red (time zero) to yellow, green, cyan, and finally blue (60 s) and combined into a single image, yielding rainbow tracks. White cells correspond to cells that showed little or no net migration. The multi-colored “stars” indicate that cells attached to the glass at one pole had rotated or inverted. The rainbow trajectory corresponds to the sequence shown in Movies S1–3. The scale bar represents 50 µm. **(B)** Graph showing gliding motility velocity of wild-type, FTN25, and FTN26. Boxplots display the median of 100 bacterial cells in one experiment. Crosses indicate mean values and horizontal lines in boxes indicate median values. Asterisks denote Student’s t-test significance compared with wild-type (* *p* < 0.05).

To examine the gliding motility of *F. collinsii* GiFuPREF103, bacterial cells were introduced into tunnel slides and observed with a phase contrast microscope. FTN25 and FTN26 mutant cells showed gliding in the reverse direction, resulting in reduced net progress compared to the wild-type strain (**Figure 3**, see **Movies S1–S3**). On comparing the average gliding velocities of 100 bacteria, the wild-type strain had a gliding velocity of 1.24 µm/s, while FTN25 and FTN26 had velocities of 0.86 and 0.82 µm/s, respectively. FTN25 and FTN26 also showed an increase in the frequency of inversions (**Figure 3A**). These findings suggest that FTN25 and FTN26 may be partially defective in motility.

### 3.5 Pep25 and Lbp26 are involved in social process of forming biofilms

**Figure 4:**
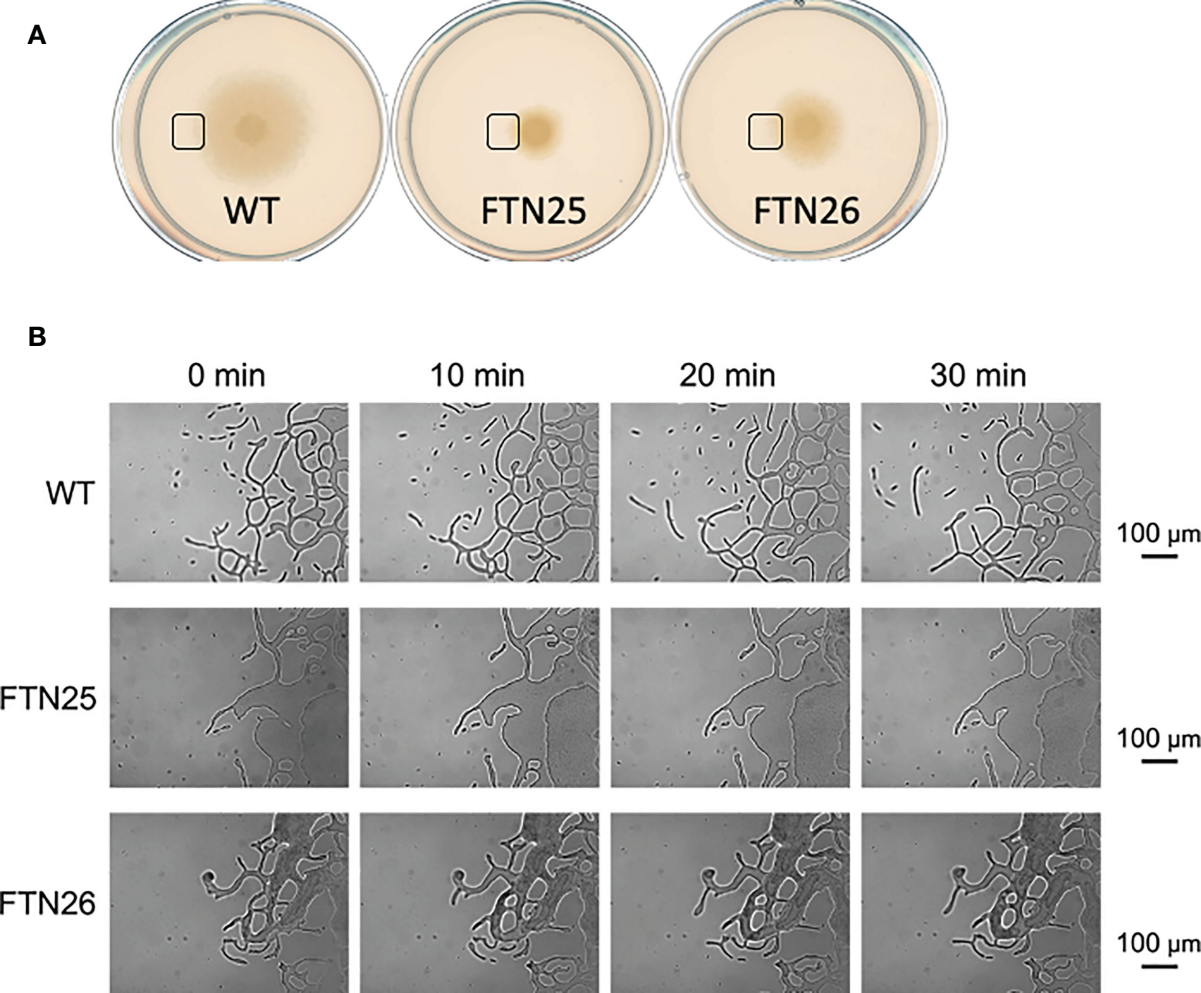
Colony spreading of *F*. *collinsii* GiFuPREF103 on agar plate. **(A)** Colony spreading of WT, FTN25, and FTN26 on MCP agar medium. **(B)** Higher magnification images of the squares in **(A)**. The movement of bacterial cells at the colony margin was observed with a phase contrast microscope. Images were recorded at 30 s intervals for 30 min. Times are shown at the top of the figure. The scale bar represents 100 µm and applies to all panels. These images correspond to Movies 4–6. In wild-type, small groups can be observed popping up from the margins of the colony and moving actively (see **Movies S4**). In contrast, in FTN25 and FTN26, the small groups observed in the wild-type strain were not observed and the colonies spread slowly (see **Movies S5, S6**).

FTN25 and FTN26 with partial defects in gliding motility also had diminished colony spreading capacity compared to that in the wild-type (**Figure 2B**). When observing the edge of wild-type *F. collinsii* GiFuPREF103 colonies under high magnification, small cell clusters were found to break away from the main colony and glide (**Figure 4**, see **Movie S4**). In contrast, in FTN25 and FTN26, such small gliding clusters away from the main wild-type colony were not observed, and the colonies spread gradually by extending dendritic protrusions at their edges (**Figure 4**, see **Movies S5, S6**). To examine this in detail, bacterial cells at the edges of mutant colonies were observed using fluorescence microscopy. For these experiments, *F. johnsoniae* was used as a surrogate for *F. collinsii* GiFuPREF103, as a strain expressing a fluorescent protein was required for observation. Observation of the edges of wild-type *F. johnsoniae* colony revealed clusters of actively gliding bacterial cells (**Movie S7**). In contrast, at the edges of *F. johnsoniae Δfjoh_0352* and *Δfjoh_0353* clusters, most bacterial cells were either still or moved back and forth, while several cells kept moving forward but were sometimes interrupted by the immobile cells (see **Movies S8, S9**). At the edge of the spreading colony, the wild-type strains exhibited fast population movement, from densely crowded areas toward less crowded areas, whereas for *Δfjoh_0352* and *Δfjoh_0353*, individual bacterial cells exhibited gliding motility, but no collective behavior was observed.

### 3.6 FTN25 and FTN26 strains show strong auto-aggregation with an increase in hydrophobicity of the cell surface

**Figure 5:**
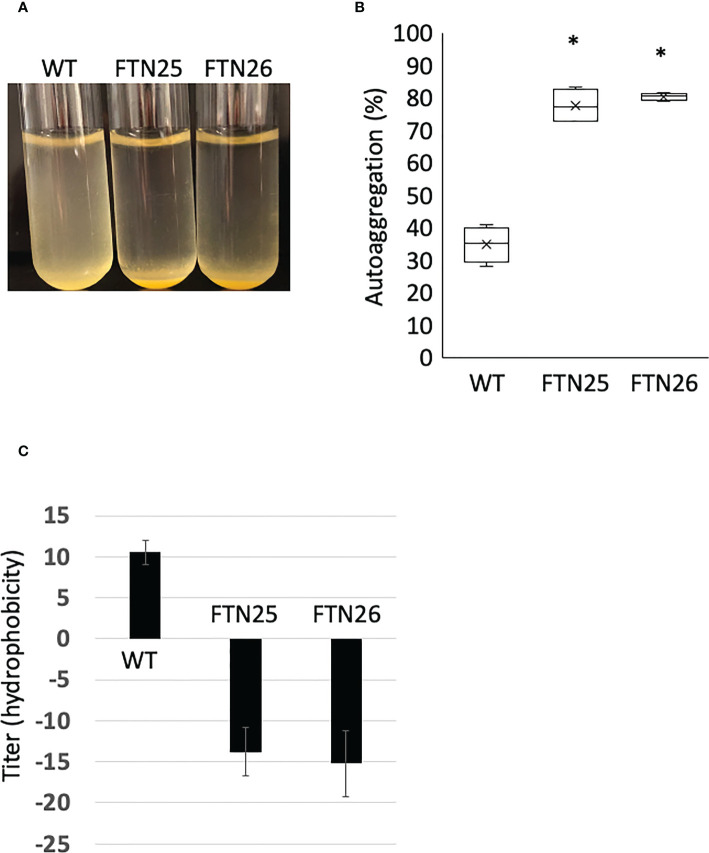
Auto-aggregation of *F*. *collinsii* GiFuPREF103. The bacterial cells were grown overnight in MCP broth and then adjusted with MCP broth to an OD_595_ of 0.6. The medium was allowed to stand for 10 min to allow the bacteria to settle. **(A)** The FTN25 and FTN26 cells auto-aggregated at the bottom of the tube, but the wild-type cells did not. **(B)** The following formula quantified the auto-aggregating property: [100 – (mean OD_595_ of supernatant/0.6)] × 100%. The auto-aggregation abilities of FTN25 and FTN26 cells were enhanced compared to that of the wild-type. Boxplots display the median of four biological replicates in one experiment. Crosses indicate mean values and horizontal lines in boxes indicate median values. Asterisks denote Student’s t-test significance compared with wild-type (* *p* < 0.05). **(C)** Hydrophobicity assay using hexadecane. Each strain standardized at OD_595_ = 1.0 in PBS was used for a hexadecane hydrophobicity assay. The mean ± SD of results from 4 independent experiments are shown (* *p* < 0.05).

Hydrophobicity, biophysical characteristics of the cell surface, affect both cell-cell and cell-surface interactions and are involved in biofilm formation in hydrophilic environments. In *F. johnsoniae* Δ*fjoh_0352*, reports have suggested that the cells are more hydrophobic and have stronger cell–cell interactions and tight connections in the liquid medium (Li et al., 2021). FTN25 and FTN26 showed stronger auto-aggregation than the wild-type (**Figures 5A, B**). Furthermore, the hydrophobicity of the bacterial surface was examined using a hexadecane assay. FTN25 and FTN26 had higher cell surface hydrophobicity than the parent strain (**Figure 5C**). These results suggest that *pep25* and *lbp26* contribute to the hydrophilicity of the bacterial cell surface.

### 3.7 Hemagglutination activity of *F. collinsii* GiFuPREF103

**Figure 6:**
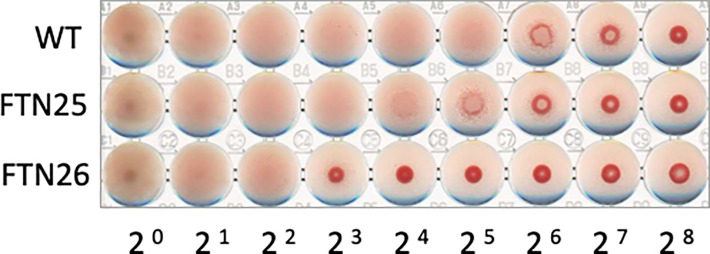
Hemagglutination ability of *F. collinsii* GiFuPREF103 cells grown in MCP broth. The washed bacteria were suspended in PBS and serial dilutions in a 2-fold series were applied to the wells of a microtiter plate from left to right and mixed with chicken erythrocyte suspension.

The hemagglutinating properties of *F. collinsii* GiFuPREF103 were further investigated. The *lpb26* mutant FTN26 showed less hemagglutination compared to the wild-type (**Figure 6**). The *pep25* mutant FTN25 showed almost the same hemagglutination as that of the wild-type strain (**Figure 6**). These results demonstrate that the *lpb26* gene contributed to cell-induced hemagglutination in *F. collinsii* GiFuPREF103. Similar results were obtained in hemagglutination tests with rabbit erythrocyte.

### 3.8 Mutants formed biofilms with enhanced microcolony growth compared to the wild-type

**Figure 7:**
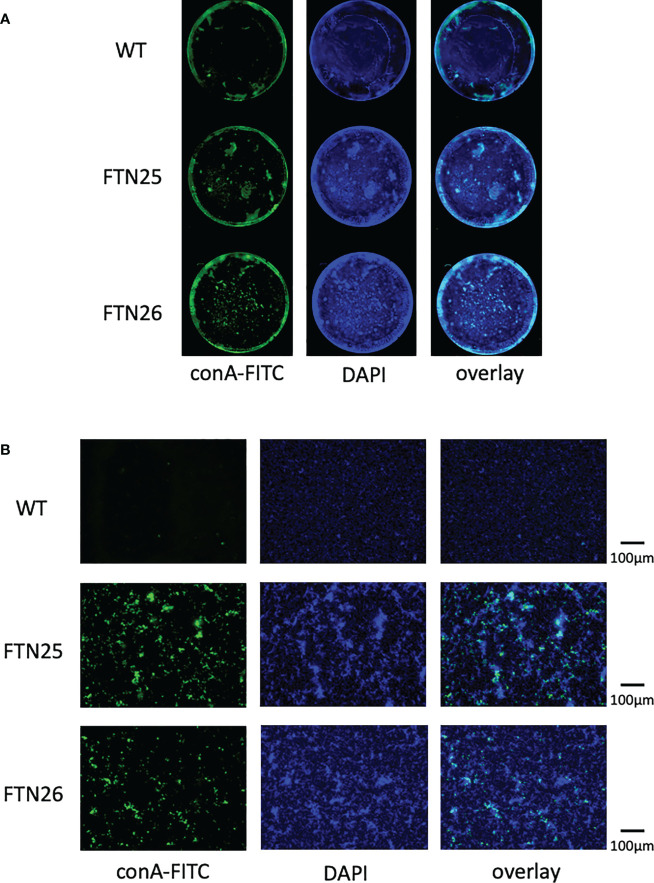
Static biofilm structure formed by *F*. *collinsii* GiFuPREF103. Each strain was cultured in 24-well glass dishes under aerobic conditions. Biofilms were treated with DAPI (blue channel) and fluorescein-bound concanavalin A (ConA-FITC) (green channel) and visualized using a BZ-X800 fluorescence microscope. A single channel and an image overlay are shown. **(A)** Images were taken using a 4× lens. Images were concatenated using BZ-X800 Analyzer software to display the entire well. **(B)** An image near the center of the well was taken using a 20× lens. The scale bar represents 100 µm. FTN25 and FTN26 formed dense, multilayered biofilms. In contrast, biofilms formed by wild-type strains were less developed and thin structures, with occasional unevenly distributed microcolony-like structures (center panel, DAPI). Strong ConA signal was observed in the biofilms formed by FTN25 and FTN26, suggesting the presence of glucose and/or mannose residues (left, green signal) and ConA signal was closely co-localized with the DAPI-stained cells (right, overlay).

Fish pathogen *F. columnare* (Cai et al., 2013) forms mature biofilms containing EPM and waterways when grown on glass slides soaked in liquid medium (Levipan and Avendano-Herrera, 2017). To determine whether *F. collinsii* GiFuPREF103 wild-type, FTN25, and FTN26 differ in biofilm formation, the strains were cultured for 24 h in glass bottom dishes and the biofilms formed were analyzed using fluorescence microscope. DAPI was used to visualize the cells. The results revealed that FTN25 and FTN26 formed dense, multilayered biofilms. In the wild-type strain, the DAPI signal was relatively uniform compared to that in the mutant strains (**Figure 7**, center panel, DAPI). To visualize the production of EPM in the biofilms, the biofilms were stained with the fluorescence (FITC)-labeled lectin ConA. Strong ConA signals were observed in the biofilms formed by FTN25 and FTN26 (**Figure 7**, left panel, ConA-FITC) and the signals were closely co-localized with the DAPI-stained cells (**Figure 7**, right panel, overlay). The other lectins such as phytohemagglutinin-L, soybean agglutinin, and wheat germ agglutinin did not interact with the wild-type or with mutants, suggesting the presence of glucose and/or mannose residues in the biofilms of the mutants.

## 4 Discussion

Fish such as salmon, trout, and other fish are hosts for bacterial infections caused by *F. psychrophilum*, resulting in significant losses to farmed fish production worldwide (Madsen and Dalsgaard, 1998). *F. columnare* is reported to attach to gill epithelial tissues, scales, and fins (Decostere et al., 1999). In addition to causing fish diseases, many *Flavobacterium* have been identified in various environmental biofilms (McBain et al., 2003; Rickard et al., 2004). *Flavobacterium* and *Chryseobacterium* were isolated from *Plecoglossus altivelis* that developed BCWD in a river or aquaculture farm in Gifu Prefecture, Japan. Among them, *F. collinsii* GiFuPREF103 was identified, which has not been reported in *Plecoglossus altivelis*. Phylogenetic tree analysis using 16S rRNA sequences confirmed that *F. collinsii* GiFuPREF103 is more closely related to the environmental microorganisms *F. johnsoniae* than to the BCWD pathogen *F. psychrophilum*. Furthermore, whole-genome sequencing revealed that *F. collinsii* GiFuPREF103 possesses complete sets of *gld*, *spr*, and *por* genes (McBride, 2019). Therefore, it is suggested that this strain glides and forms spreading colonies through a gliding motility mechanism similar to that of *F. johnsoniae* (McBride and Braun, 2004). T9SS is conserved in nonpathogenic environmental microbes of phylum *Bacteroidota* such as *C. hutchinsonii* and *F. johnsoniae* (McBride and Zhu, 2013). As both the bacteria have the ability to digest macromolecules such as cellulose and chitin and the secretion of chitinase and cellulase requires T9SS, therefore this system serves as a tool for migration and nutrition acquisition by the bacteria (Zhu and McBride, 2014; Kharade and McBride, 2014; Yang et al., 2016).

Pep25 and Lbp26 showed similarity to Wza and Wzz, respectively. Wza belongs to the large family of outer membrane polysaccharide export proteins. The *wza* mutant of *Riemerella anatipestifer*, a duck infectious pathogen causing serositis, lacks the capsular polysaccharide, but still harbors the LPS-O-antigen. Furthermore, the mutant strain was found to be more hydrophobic, showed stronger auto-aggregation, and underwent increased biofilm formation than the parental strain, similar to that with FTN25 and FTN26 (Yi et al., 2017). In *Klebsiella pneumoniae*, the capsule was visualized as high-molecular weight material, and the *wza*-mutant strain lacked the capsular polysaccharide. Wzz is important for regulating O-antigen polymer chain length (Franco et al., 1996). In *P. gingivalis*, the length of the polymer chain of O-LPS, as well as A-LPS, is regulated by Wzz (Shoji et al., 2013). In *Flavobacterium* spp., the anionic lipopolysaccharide has not yet been found. It is suggested that FTN25 and FTN26 might also be affected by the synthesis and polymer chain assembly of O-LPS and/or the capsular polysaccharide.

*Flavobacterium* spp. do not have flagella or type IV pili, a conventional motility structures, and move on solid surfaces using gliding motility (Nakane et al., 2013). T9SS cargo proteins have a conserved domain at the C-terminus called the CTD signal that allows them to penetrate the outer membrane using the T9SS (Seers et al., 2006; Chen et al., 2011; Veith et al., 2013). Two types of CTD signals have been defined so far, type A and type B, differing in some of the T9SS components (Kulkarni et al., 2017; Gorasia et al., 2022). Type A CTD proteins are anchored to the bacterial cell surface *via* linkage to the anionic lipopolysaccharide, but it is not known how type B CTD proteins are anchored to the bacterial cell surface. SprB, the major motility adhesin in *F. johnsoniae*, was reported to have a type B CTD secreted by T9SS (Kulkarni et al., 2019). In this study, SprB was found to be localized to the bacterial surface in FTN25 and FTN26, similar to that in the wild-type strain. At the edge of the spreading colony, the wild-type exhibited fast cell population flow from dense crowds toward less crowded areas. Interestingly, at the colony-spreading margin of Δ*fjoh_0352* and Δ*fjoh_0353*, we observed many stationary bacterial cells and individual cells gliding without contact with other individuals. FTN25 and FTN26 showed a slower glide speed and more frequent changes of direction than wild-type. Further analysis of the correlation between gliding speed and directional rotation frequency is needed. These factors may be involved in decreased colony-spreading ability.

A microbial biofilm is a community of surface-attached microorganisms embedded in self-produced EPM. Biofilm formation begins with the initial attachment of bacteria to surfaces, leading to the formation of microcolonies and maturation of the microcolonies into a three-dimensional structure surrounded and stabilized by an EPM. Adhesion is a complex multi-step process that can be subdivided into attraction, adhesion, and aggregation stages (O'Toole et al., 2000). The biofilm formation capacity of FTN25 and FTN26 was higher than that of wild-type, in this study. ConA signals were observed in biofilms formed by FTN25 and FTN26, indicating the presence of glucose and/or mannose residues, which form major components of EPM. The ConA signal co-localized with the DAPI stained cells. On the other hand, a strong ConA signal was not observed in biofilms formed by wild-type cells. The presence of gliding bacteria in the aquatic environment and their ability to adhere to various substrates suggests that gliding bacteria may be members of microbial biofilms (Burchard and Sorongon, 1998), since transient attachment is required for the functioning of their motility mechanism. Gliding motility-deficient mutations in *F. psychrophilum* have been reported to have contradictory properties between gliding motility and biofilm formation (Alvarez et al., 2006). In this study, FTN25 and FTN26 showed an increase in biofilm formation capacity but a decrease in gliding motility speed.

Overall cell hydrophobicity, auto-aggregation, and coaggregation are important for colony and biofilm formation in a fluid environment (Rickard et al., 2004). It is generally accepted that higher hydrophobicity increases adhesion and lower hydrophobicity decreases adhesion (Van Loosdrecht et al., 1987). In this study, the wild-type strain formed relatively uniform colonies, whereas two mutants exhibited massive microcolonies in the submerged environment on the glass. In particular, the *lbp26* mutant formed fewer but massive microcolonies. The results of these mutants were similar to those of the *F. johnsoniae fjoh_0352* mutant (Li et al., 2021). These results suggested that the mutant cells were more hydrophobic, had stronger cell-cell interactions and binding within and between microcolonies. Aggregation is the process by which genetically distinct bacteria adhere to each other *via* specific molecules, and the interaction of aggregation is enhanced by increase in hydrophobicity, which is stronger than co-aggregation (Rickard et al., 2004). ConA was rarely found at the margins of the microcolonies and was bound to the center of the microcolonies. ConA-responsive EPMs were abundant in FTN25 and FTN26, which may contribute to the hydrophobicity and aggregation of the bacteria. On the other hand, at the colony margins, the wild-type strain moved using collective motility, and the mutant strain showed reduced social motility and movement was by individual sliding movements. These results suggest that bacterial surface glycosylation affect collective motility.

Aggregation in *F. psychrophilum* strains is mediated by growth-phase-dependent lectin–glycan interactions, which are optimal but regulated in stationary phase culture (Møller et al., 2003). Therefore, determining the aggregation partners in *Flavobacterium* sp. isolates may indirectly reveal the cell surface characteristics and factors involved in the establishment of biofilm relationships with neighboring organisms. Pep25 and Lbp26 are involved in the regulation of bacterial cell surface properties, which play an important role in the attachment of bacteria to surfaces and the formation of biofilms. Biofilm formation is a complex process regulated by several different factors. Our data suggested bacterial surface properties is an important factor influencing biofilm formation. However, the results of this study were obtained *in vitro*, and *in vivo* experiments should be conducted in the future. Gliding bacteria have the ability to transport other non-motile bacteria, and their motility allows them to spread through biofilms (Shrivastava et al., 2018). This suggests that even non-pathogenic gliding bacteria may be involved in the transport and colonization of other pathogenic bacteria and in developing pathogenicity in mixed infections. These findings indicate that bacterial surface properties and gliding motility may be an important factor in the biofilm formation involving multiple bacterial species and mixed infections.

## Data availability statement

The datasets presented in this study can be found in online repositories. The names of the repository/repositories and accession number(s) can be found below: https://www.ddbj.nig.ac.jp/, BOVI01000001–BOVI01000189.

## Author contributions

YK, KO, RF, YN, CS, MN, and KS designed and performed experiments and analyzed data. YK and KS wrote the main manuscript. CS, TT, MN, and TK provided important comments and suggestions that helped improve the manuscript. All authors contributed to the article and approved the submitted version.
