# Supplementary material for: Transposon mutagenesis and genome sequencing identify two novel, tandem genes involved in the colony spreading of Flavobacterium collinsii, isolated from an ayu fish, Plecoglossus altivelis
Source: Front Cell Infect Microbiol. 2023 Feb 10;13:1095919. doi: 10.3389/fcimb.2023.1095919 (PMC9950754; doi:10.3389/fcimb.2023.1095919)
Supplement: Supplementary file 1 [file Table_1.docx]

Supplementary Material

**Transposon mutagenesis and genome sequencing identify two novel, tandem genes involved in colony spreading of *Flavobacterium collinsii*, isolated from an ayu fish, *Plecoglossus altivelis***

**Authors:** Yoshio Kondo, Kenichi Ohara, Ryoji Fujii, Yudai Nakai, Chikara Sato, Mariko Naito, Takayuki Tsukuba, Tomoko Kadowaki, Keiko Sato

# Supplementary Figures:


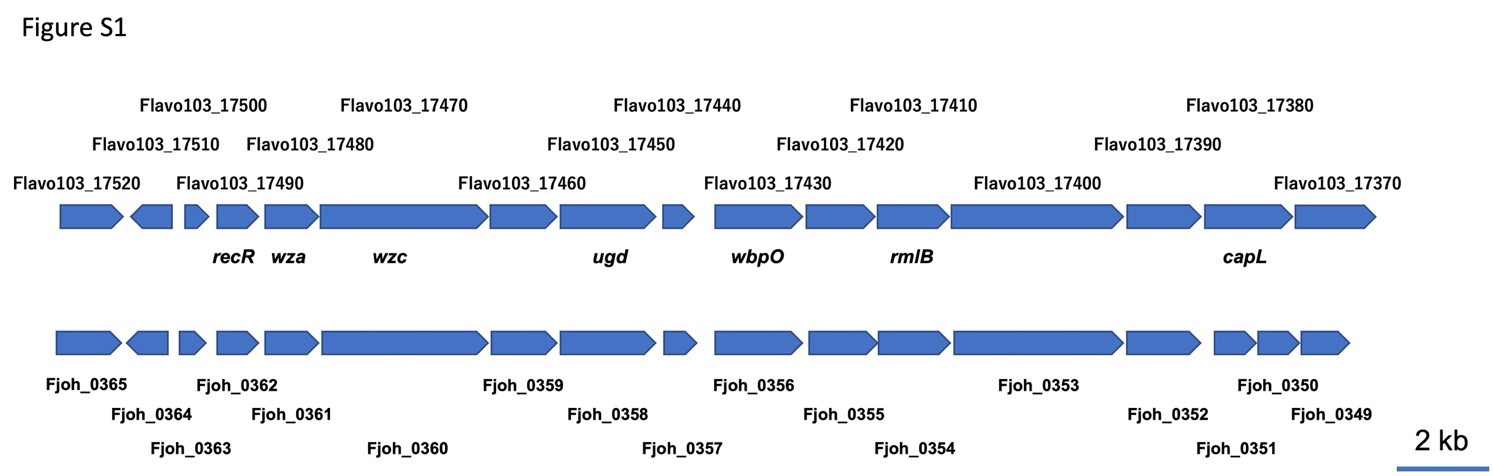


**Figure S1. Synteny maps for conserved regions including *pep25* and *lbp26* in *F. collinsii* GiFuPREF103 and *F. johnsoniae* UW101.**

Each gene is represented by an arrow indicating the direction of transcription. The top arrow represents *F. collinsii* GiFuPREF103 and the bottom arrow represents *F. johnsoniae* UW101. Scale bar represents 2 kb.


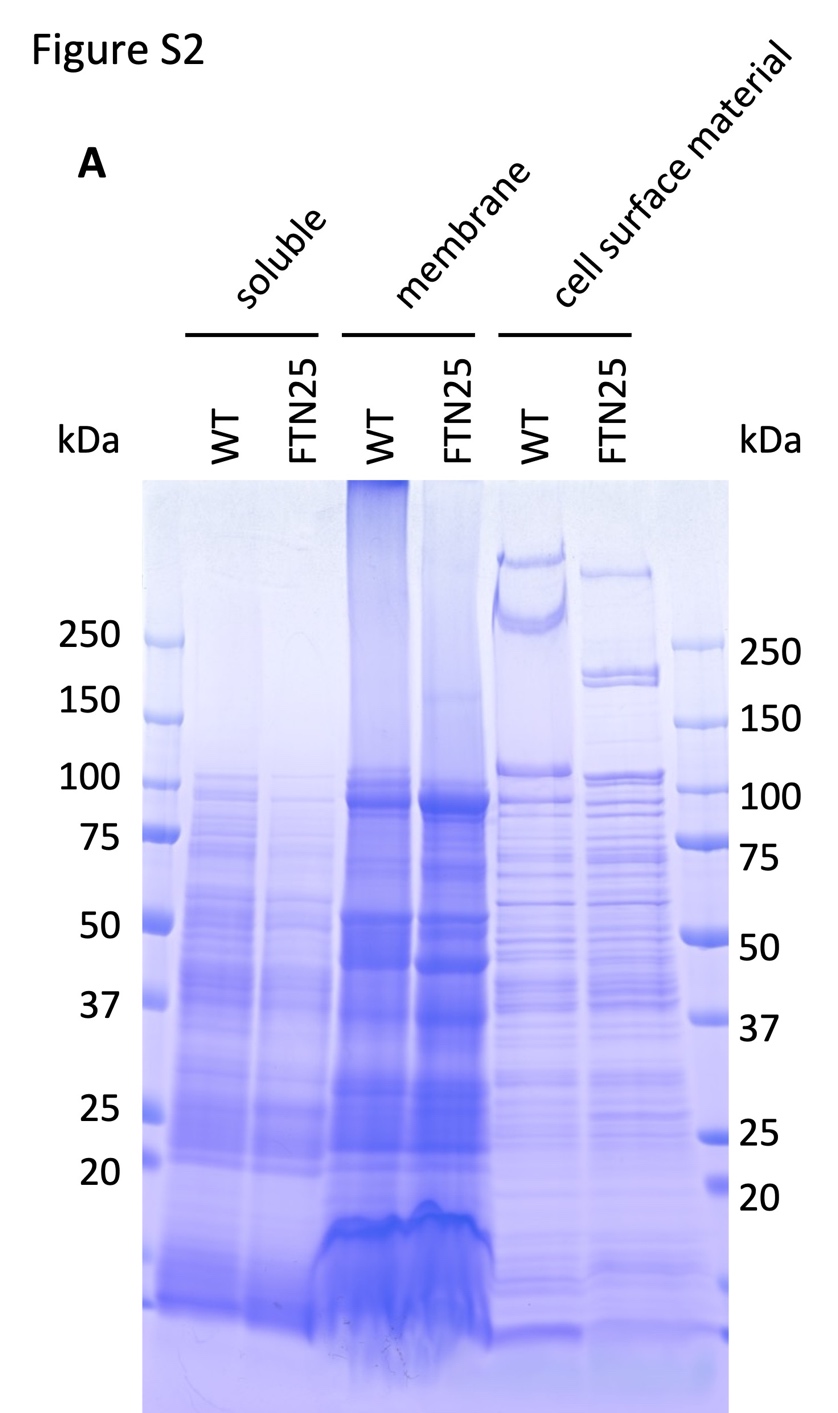

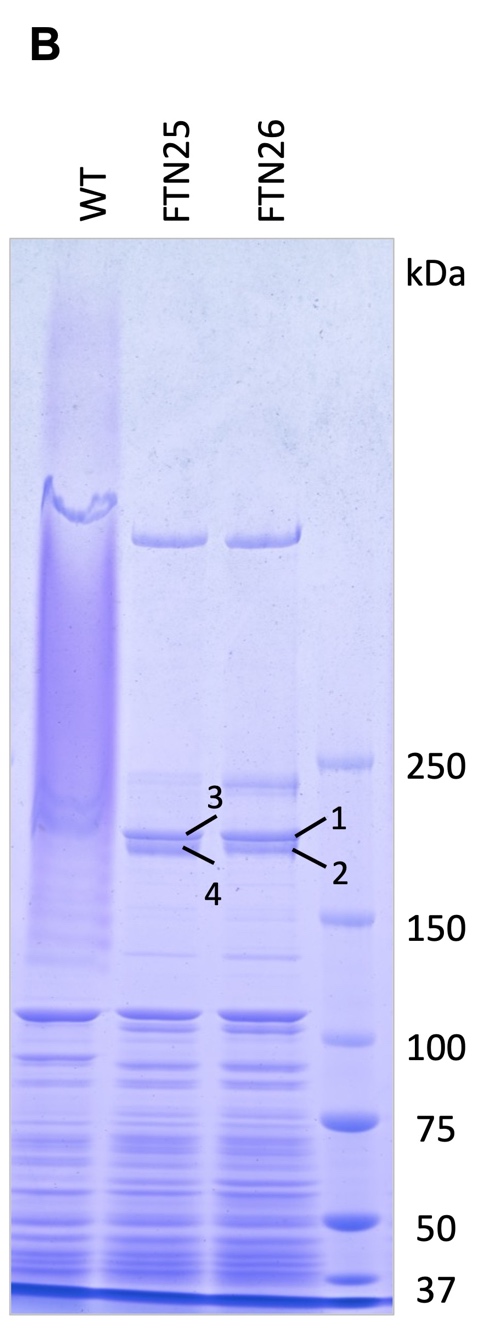

**Figure S2. SDS-PAGE analysis and mass spectrometry of bacterial surface proteins**

(A) The wild-type and FTN25 mutant strains were fractionated into soluble (cytoplasm/periplasm), membrane, cell surface material fractions and then subjected to 5%–20% gradient SDS-PAGE. (B)Extracted cell surface materials were separated on 3%–10% gradient SDS-PAGE and stained with Coomassie blue. The fractional marker location is indicated on the right side. Protein bands 1–4 were analyzed by peptide mass fingerprinting. Protein bands 1 and 3 were Flavo103_03160 (hypothetical protein) and protein bands 2 and 4 were Flavo103_21710 (hypothetical protein, type A CTD protein). However, bands of macromolecules larger than 250 kDa could not be identified by mass spectrometry.

**
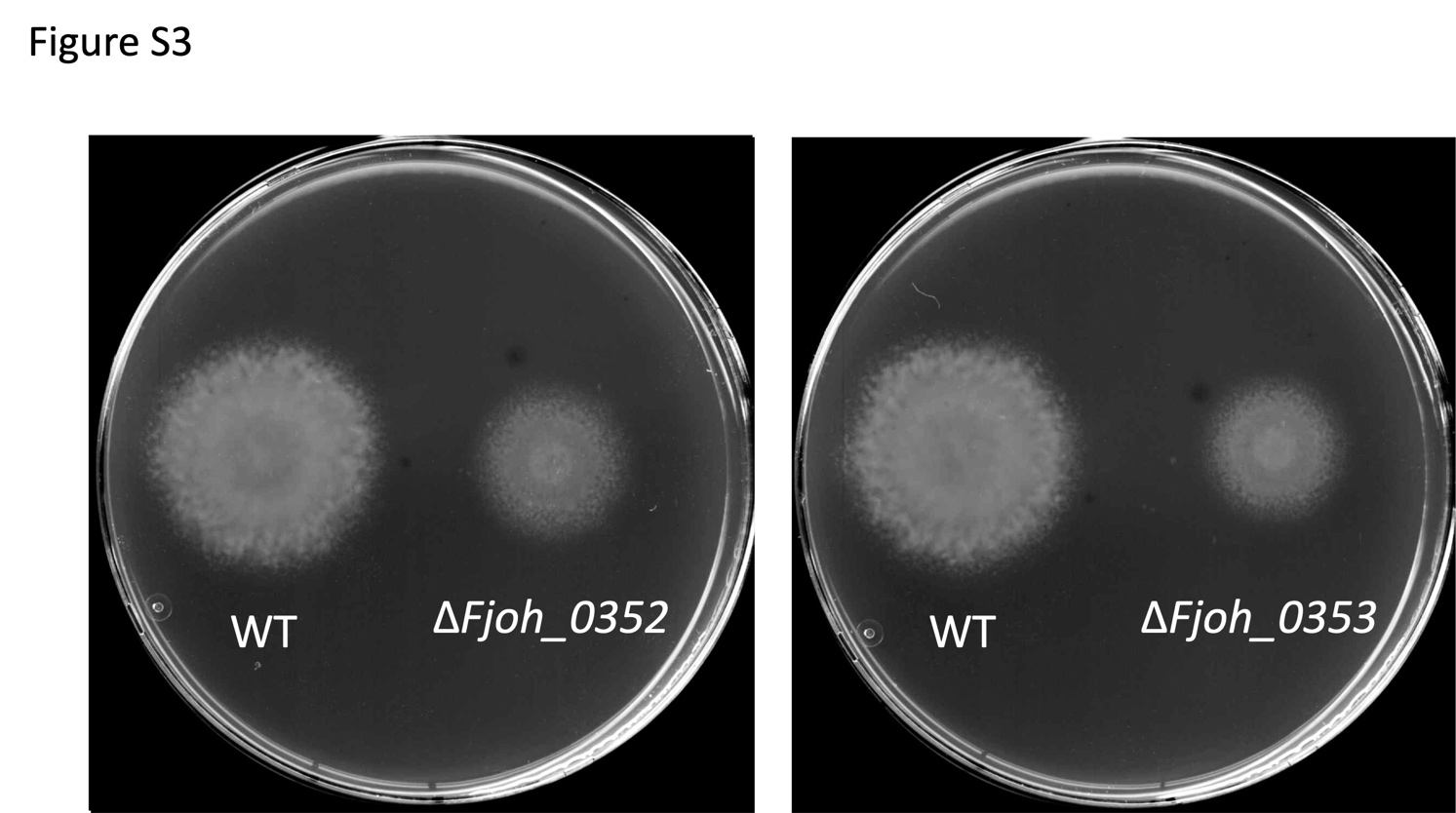
**

**Figure S3. Colony spreading of *F. johnsoniae.***

We tried to produce *Flavobacterium* sp. GiFuPREF103 strains expressing GFP to observe colony margins through fluorescence microscopy; unfortunately, we were unable to obtain them. Therefore, we experimented with *F. johnsoniae*, a closely related species that can be genetically manipulated, and in *F. johnsoniae*, as in *Flavobacterium* sp. GiFuPREF103, colony spreading was suppressed in ∆*Fjoh_0352* or ∆*Fjoh_0353*. Observation of the colony margins using a fluorescence microscope with a 100× lens revealed actively gliding bacterial cells in the wild-type strain in clusters (Movie S7). In contrast, in the ∆*Fjoh_0352* or ∆*Fjoh_0353* strains, many bacterial cells were observed without gliding (Movie S8 and S9).


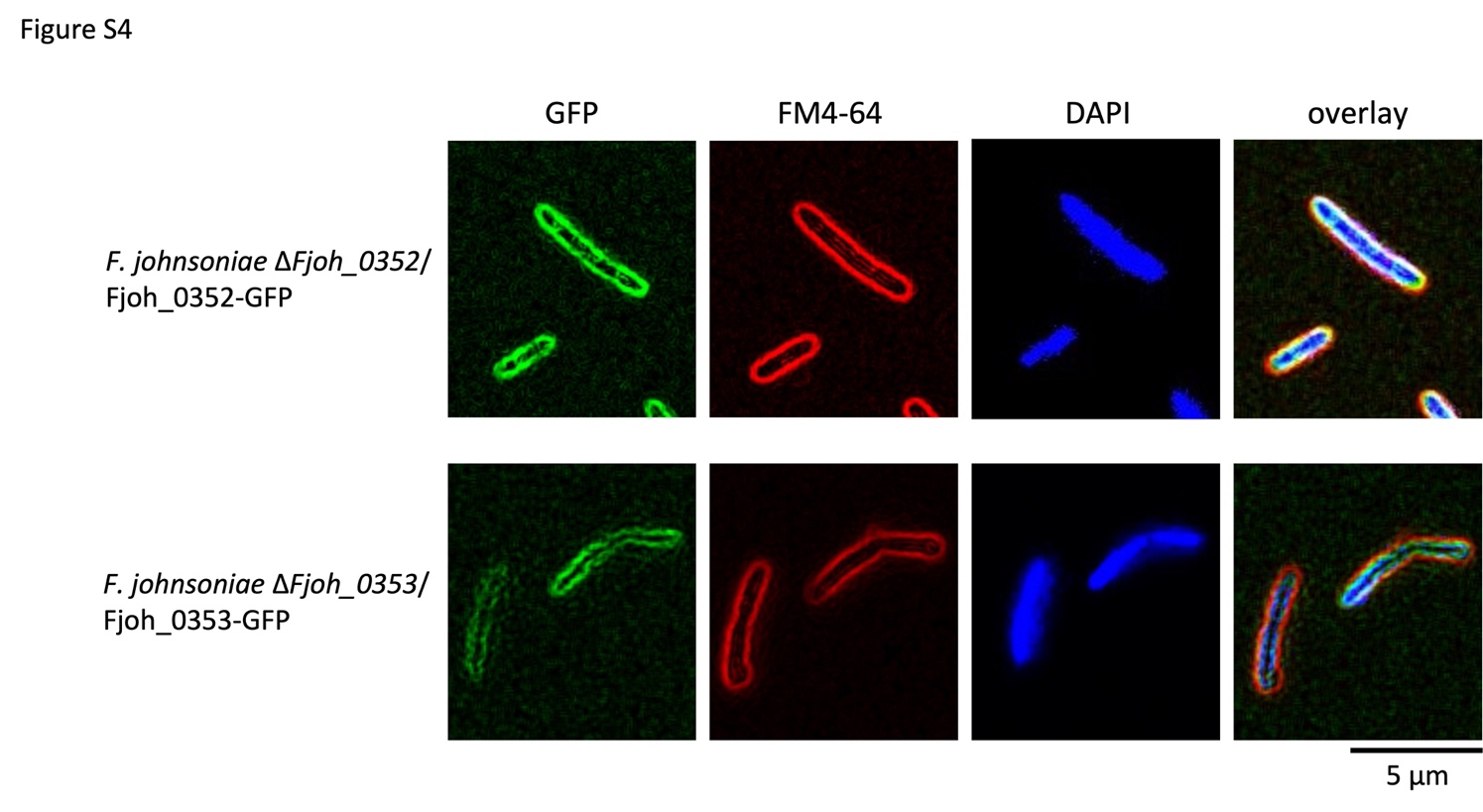


**Figure S4. Subcellular localization of Fjoh_0352 and Fjoh_0353.**

Cells were examined microscopically to locate Fjoh_0352 and Fjoh_0353. DAPI and FM4-64 were used to stain the DNA and cell membrane, respectively. All images were taken at 100× magnification. The scale bar represents 5 µm.


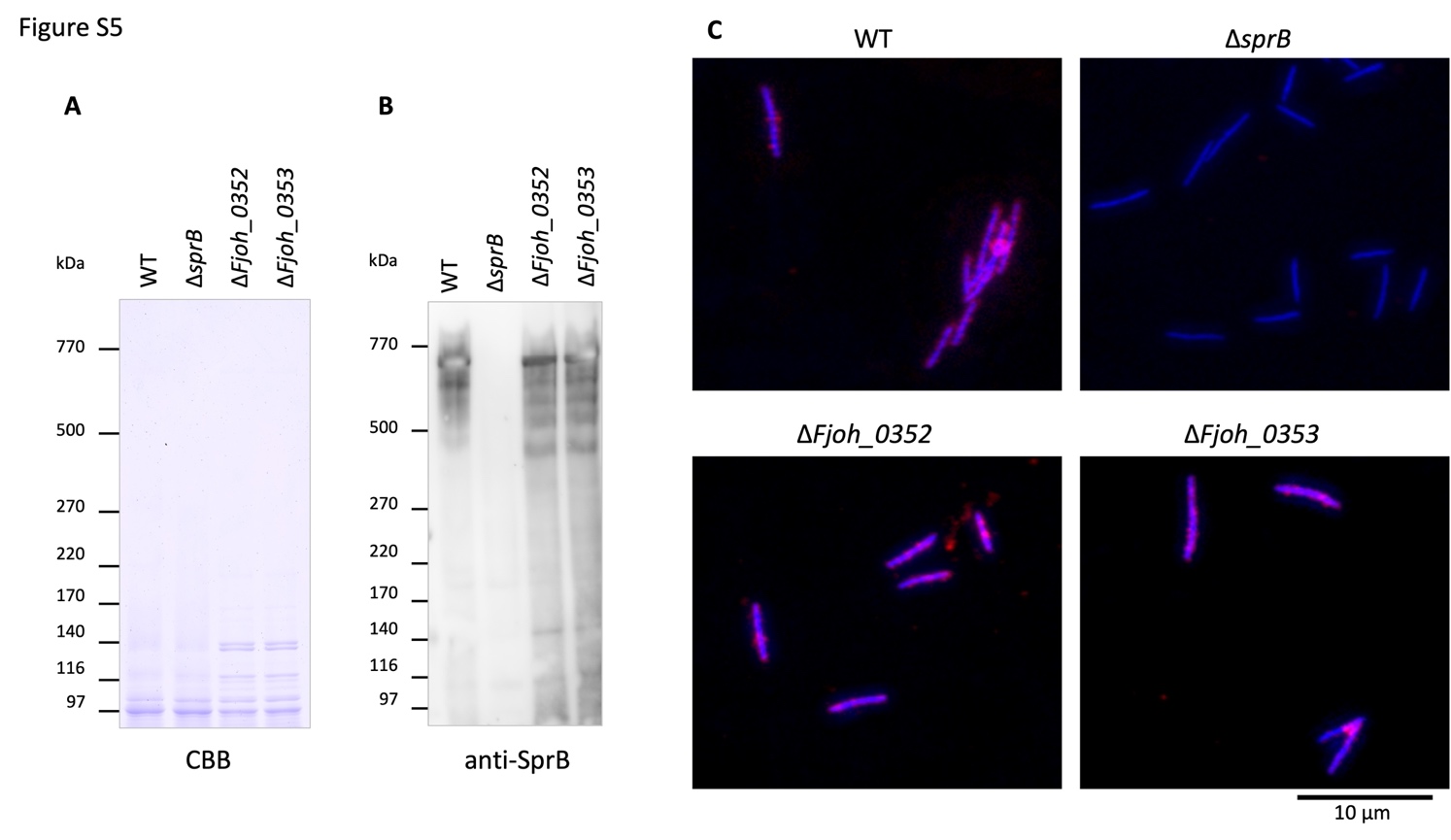


**Figure S5. Glycosylation of *F. johnsoniae* SprB and its localization on the bacterial surface.**

SDS-PAGE of the whole cell proteins extracted from *F. johnsoniae*. CJ1827, ∆sprB, ∆Fjoh_0352, and ∆Fjoh_0353 were separated on 3%–10% gradient gels and stained with (A) Coomassie blue, (B) immunoblotted with anti-SprB antibody The location of the fractionation markers is shown on the left side of each image. (C) Detection of surface-localized SprB using immunofluorescence microscopy; *F. johnsoniae* CJ1827, ∆*sprB*, ∆*Fjoh_0352*, and ∆*Fjoh_0353* cells were exposed to anti-sprB antibodies. Subsequent exposure to a secondary antibody fused to Alexa Fluor 488 and observation via fluorescence microscopy detected SprB located on the cell surface. Bacterial cells were also stained with DAPI and superimposed on the SprB signal image to observe localization in *F. johnsoniae.*

# Supplementary Tables:

**Table S1. General genomic features of *Flavobacterium* collinsii GiFuPREF103**

| **Table S1. General genomic features of*Flavobacterium* *collinsii* GiFuPREF103** | |
| --- | --- |
| Total Sequence Length (bp): | 5,586,653 |
| Number of contigs: | 189 |
| Longest Sequences (bp): | 600,566 |
| N50 (bp): | 457,314 |
| GC content (%): | 35 |
| Number of CDSs: | 4,567 |
| Average Protein Length: | 344 |
| Coding Ratio (%): | 84 |
| Number of rRNAs: | 3 |
| Number of tRNAs: | 57 |
| Number of CRISPRs: | 1 |

| **Table S2. Strain name and accession number used in 16S RNA sequence-based phylogenetic tree phylogenetic analysis** | |
| --- | --- |
| accession number | bacterial strain |
| NR_104962.1 | *Flavobacterium acidificum* strain LMG 8364 |
| NR_112819.1 | *Flavobacterium algicola* strain TC2 |
| NR_042998.2 | *Flavobacterium antarcticum* DSM 19726 strain AT1026 |
| NR_118482.1 | *Flavobacterium aquatile* strain LMG 4008 |
| NR_158093.1 | *Flavobacterium ardleyense* strain A2-1 |
| NR_145938.1 | *Flavobacterium buctense* strain T7 |
| NR_044198.1 | *Flavobacterium cheniae* strain NJ-26 |
| LC744069 | *Flavobacterium collinsii* GiFuPREF103 |
| NR_145952.1 | *Flavobacterium collinsii* strain 983-08 |
| NR_118575.1 | *Flavobacterium columnare* NBRC 100251 = ATCC 23463 |
| NR_117428.1 | *Flavobacterium dongtanense* strain LW30 |
| NR_134034.2 | *Flavobacterium faecale* strain WV33 |
| NR_112816.1 | *Flavobacterium frigoris* strain NBRC 102678 |
| NR_025538.1 | *Flavobacterium gelidilacus* strain R-8899 |
| NR_074455.1 | *Flavobacterium johnsoniae* strain UW101 |
| NR_117492.1 | *Flavobacterium koreense* strain ARSA-42 |
| NR_133745.1 | *Flavobacterium lacus* strain NP180 |
| NR_024787.1 | *Flavobacterium limicola* strain ST-82 |
| NR_145939.1 | *Flavobacterium maris* strain KMM 9535 |
| NR_104505.1 | *Flavobacterium ponti* strain GSW-R14 |
| NR_115302.1 | *Flavobacterium psychrophilum* DSM 3660 = ATCC 49418 |
| NR_043000.1 | *Flavobacterium segetis* strain AT1048 |
| NR_044804.1 | *Flavobacterium tegetincola* strain A103 |
| NR_133749.1 | *Flavobacterium tructae* strain 435-08 |
| NR_042999.1 | *Flavobacterium weaverense* strain AT1042 |
| NR_109689.1 | *Flavobacterium yanchengense* strain hg |

**Table S2. Strain name and accession number used in 16S RNA sequence-based phylogenetic tree phylogenetic analysis**

**Table S3. Oligonucleotides used in this study**

| **Table S3. Oligonucleotides used in this study** | | |
| --- | --- | --- |
| Primer | Sequence (5'–3') | Reference or source |
| 27F | 5'-AGAGTTTGATCCTGGCTCAG-3' | Lane et al |
| 1500R | 5'-TACCTTGTTACGACTT-3' | The Japanese Pharmacopoeia 18th edition |
| 800F | 5'-GGATTAGATACCCTGGTA-3' | The Japanese Pharmacopoeia 18th edition |
| 800R | 5'-TACCAGGGTATCTAATCC-3' | The Japanese Pharmacopoeia 18th edition |
| AR8 | 5’-GGCCACGCGTCGACTAGTAC(N)10GATGC-3’ | Ichimura et al |
| AR2 | 5’-GGCCACGCGTCGACTAGTAC-3’ | Ichimura et al |
| mariner-A | 5’-CACAACCGACAACTTGAACATTTCGGGC-3’ | Ichimura et al |
| mariner-B | 5’-CCCTTGCCTGCCCCAATATCTAAAACCGTA-3’ | Ichimura et al |
| mariner-S | 5’-GTTGCAGATGAGCAAACATATAACCGAGGA-3’ | Ichimura et al |
| F0353-UF-BamHI | 5’-GGATCCGCAGATAAAGTGATGGTTGTCGG-3’ | This study |
| F0353-UR-SalI | 5’-GTCGACGAACGTATATTATTTTTTTCAT-3’ | This study |
| F0353-DF-SalI | 5’-GTCGACTTGGCATTGTTAATTGTAACTGC-3’ | This study |
| F0353-DR-SphI | 5’-GCATGCTTCATAGTGCCAATCACGTGCATC-3’ | This study |
| F0353-coF-BamHI | 5’-GGATCCATGAAAAAAATAATATACGTTCT-3’ | This study |
| F0353-GR-NotI | 5’-GCGGCCGCATTTAAAAGCAGTTACAATTAA-3’ | This study |
| F0352-UF-BamHI | 5’-GGATCCAATAGCTATTGAGTATGTTGGTC-3’ | This study |
| F0352-UR-SalI | 5’-GTCGACCGATATTTCATCGTTTTCAATAAG-3’ | This study |
| F0352-DF-SalI | 5’-GTCGACAGTTAGAAGGTTGTTTAAGCAAG-3’ | This study |
| F0352-DR-SphI | 5’-GCATGCGCAATAGCCCTATTACTTATAGGG-3’ | This study |
| F0352-coF-BamHI | 5’-GGATCCATGAATGATAAACTTATTGAAAACGA-3’ | This study |
| F0352-GR-NotI | 5’-GCGGCCGCAACTAAACAATACTTGCTTAAAC-3’ | This study |

**References**

Lane DJ. 16S/23S rRNA sequencing. Nucleic acid techniques in bacterial systematics. 1991:115-75.

“Japanese Pharmacopoeia 18th Edition,” Rapid Identification of Microorganisms Based on Molecular Biological Method <G4-7-160>, 2021, pp. 2695.

Ichimura M, Uchida K, Nakayama‐Imaohji H, Hirakawa H, Tada T, Morita H, et al. Mariner‐based transposon mutagenesis for Bacteroides species. Journal of basic microbiology. 2014;54(6):558-67.

**Table S4. Distribution of T9SS-related genes**

| **Table S4. Distribution of T9SS-related genes** | | | | |
| --- | --- | --- | --- | --- |
| protein name or locus tag | predicted role | F. psy | F. joh | SP103 |
| GldA | ATP-binding cassette (ABC) transporter | FP0252 | Fjoh_1516 | Flavo103_24950 |
| GldF |  | FP1089 | Fjoh_2722 | Flavo103_36610 |
| GldG |  | FP1090 | Fjoh_2721 | Flavo103_36620 |
| GldB | Unknown | FP2069 | Fjoh_1793 | Flavo103_06660 |
| GldD | Unknown | FP1663 | Fjoh_1540 | Flavo103_25150 |
| GldH | Unknown | FP0024 | Fjoh_0890 | Flavo103_19080 |
| GldI | peptidyl-prolyl cis-trans isomerase | FP1892 | Fjoh_2369 | Flavo103_13590 |
| GldJ | Unknown | FP1389 | Fjoh_1557 | Flavo103_25360 |
| GldK | core components of the T9SS | FP1973 | Fjoh_1853 | Flavo103_07080 |
| GldL |  | FP1972 | Fjoh_1854 | Flavo103_07090 |
| GldM |  | FP1971 | Fjoh_1855 | Flavo103_07100 |
| GldN/O |  | FP1970 | Fjoh_1856 | Flavo103_07110 |
| SprA | OM pore | FP2121 | Fjoh_1653 | Flavo103_05220 |
| SprB | motility adhesin | FP0016 | Fjoh_0979 | Flavo103_19710 |
| SprE | Unknown | FP2467 | Fjoh_1051 | Flavo103_20470 |
| SprF | secretion of SprB | FP0017 | Fjoh_0978 | Flavo103_19700 |
| SprT | Unknown | FP0326 | Fjoh_1466 | Flavo103_24360 |
| PorV | Shuttles secreted protein | FP1387 | Fjoh_1555 | Flavo103_25340 |
| PorU | Sortase | FP1388 | Fjoh_1556 | Flavo103_25350 |
| Fjoh_2755 (PorQ*) | Unknown | FP1713 | Fjoh_2755 | Flavo103_43470 |
| Fjoh_0707 (PorZ*) | modification of secreted proteins | FP0270 | Fjoh_0707 | Flavo103_35190 |
| RemA | additional motility adhesin | FP1959 | Fjoh_0808 | Flavo103_35960 |
| RemG | Unknown | FP0013 | Fjoh_0983 | Flavo103_19750 |

F. psy, *Flavobacterium psychrophilum* JIP02/86; F. joh, *Flavobacterium johnsoniae* UW101;

SP103, *Flavobacterium collinsii* GiFuPREF103;

* *P. gingivalis* protein name

**Table S5. Table S5. C-terminal domain (CTD) proteins, porP and porP-like proteins predicted using genomic information**

| **Table S5. C-terminal domain (CTD) proteins, porP and porP-like proteins predicted using genomic information** | |
| --- | --- |
| type A CTD containing protein |  |
| Flavo103_02750 | hypothetical protein |
| Flavo103_02890 | hypothetical protein |
| Flavo103_04020 | hypothetical protein |
| Flavo103_07580 | hypothetical protein |
| Flavo103_09270 | hypothetical protein |
| Flavo103_12210 | hypothetical protein |
| Flavo103_13250 | hypothetical protein |
| Flavo103_13320 | hypothetical protein |
| Flavo103_14890 | hypothetical protein |
| Flavo103_15080 | hypothetical protein |
| Flavo103_15910 | hypothetical protein |
| Flavo103_16380 | hypothetical protein |
| Flavo103_19030 | hypothetical protein |
| Flavo103_21070 | hypothetical protein |
| Flavo103_21710 | hypothetical protein |
| Flavo103_21850 | hypothetical protein |
| Flavo103_22270 | hypothetical protein |
| Flavo103_26680 | hypothetical protein |
| Flavo103_29580 | hypothetical protein |
| Flavo103_29900 | hypothetical protein |
| Flavo103_33860 | T9SS C-terminal target domain-containing protein |
| Flavo103_35190 | ABC transporter substrate-binding protein |
| Flavo103_35890 | hypothetical protein |
| Flavo103_36410 | hypothetical protein |
| Flavo103_37430 | hypothetical protein |
| Flavo103_38300 | hypothetical protein |
| Flavo103_38470 | hypothetical protein |
| Flavo103_39790 | hypothetical protein |
| Flavo103_41410 | hypothetical protein |
| Flavo103_41760 | hypothetical protein |
| Flavo103_41810 | hypothetical protein |
| Flavo103_42340 | hypothetical protein |
| Flavo103_45440 | hypothetical protein |
|  |  |
| type B CTD containing protein |  |
| Flavo103_01410 | T9SS C-terminal target domain-containing protein |
| Flavo103_12530 | hypothetical protein |
| Flavo103_19710 | hypothetical protein (*F. johnsoniae sprB* orthologue) |
| Flavo103_30550 | hypothetical protein |
| Flavo103_41820 | hypothetical protein |
|  |  |
| porP and porP-like protein |  |
| Flavo103_05150 | membrane protein |
| Flavo103_05460 | membrane protein |
| Flavo103_12540 | membrane protein (porP) |
| Flavo103_19700 | membrane protein (sprF) |
| Flavo103_19720 | hypothetical protein |
| Flavo103_26370 | membrane protein |
| Flavo103_29640 | membrane protein |
| Flavo103_30540 | membrane protein |
| Flavo103_38310 | membrane protein |
| Flavo103_39110 | membrane protein |
| Flavo103_41680 | membrane protein |
| Flavo103_41830 | hypothetical protein |
| Flavo103_43360 | membrane protein |
| Flavo103_44060 | membrane protein |
| Flavo103_44090 | hypothetical protein |

# Movies:

**Movie S1: *Flavobacterium* sp. GiFuPREF103 wild type-strain gliding on glass surface**

**Movie S2: *Flavobacterium* sp. GiFuPREF103 FTN25 gliding on glass surface**

**Movie S3: *Flavobacterium* sp. GiFuPREF103 FTN26 gliding on glass surface**

**Movie S4: Colony spreading of *Flavobacterium* sp. GiFuPREF103 wild-type strain**

**Movie S5: Colony spreading of *Flavobacterium* sp. GiFuPREF103 FTN25**

**Movie S6: Colony spreading of *Flavobacterium* sp. GiFuPREF103 FTN26**

**Movie S7: Colony spreading of *F. johnsoniae* CJ1827 (100× magnification)**

**Movie S8: Colony spreading of *F. johnsoniae* ∆*Fjoh_0352* (100× magnification)**

**Movie S9: Colony spreading of *F. johnsoniae* ∆*Fjoh_0353* (100× magnification)**
